# Discovery of genes that positively affect biomass and stress associated traits in poplar

**DOI:** 10.3389/fpls.2024.1468905

**Published:** 2024-10-18

**Authors:** Tatyana Georgieva, Yordan Yordanov, Elena Yordanova, Md Rezaul Islam Khan, Kaiwen Lyu, Victor Busov

**Affiliations:** ^1^ College of Forest Resources and Environmental Science, Michigan Technological University, Houghton, MI, United States; ^2^ Department of Biological Sciences, Eastern Illinois University, Charleston, IL, United States

**Keywords:** activation tag, poplar, fasciclin like gene, patatin, drought stress, gene discovery tools

## Abstract

Woody biomass serves as a renewable resource for various industries, including pulp and paper production, construction, biofuels, and electricity generation. However, the molecular mechanisms behind biomass traits are poorly understood, which significantly curtails the speed and efficiency of their improvement. We used activation tagging to discover genes that can positively affect tree biomass-associated traits. We generated and screened under greenhouse conditions a population of 2,700 independent activation tagging lines. A total of 761 lines, which had significantly and positively affected at least one biomass-associated trait, were discovered. The tag was positioned in the genome for forty lines which were affected in multiple traits and activation of proximal genes validated for a subset. For two lines we fully recapitulated the phenotype of the original lines through overexpression. Moreover, the overexpression led to more pronounced and additional improvements, not observed in the original lines. Importantly, the overexpression of a Fasciclin-like gene (PtaFLA10) and a Patatin-like gene (PtaPAT) was found to substantially improve biomass, with a 40% increase in dry-stem weight, and enhance drought tolerance, respectively. Additionally, PtaPAT overexpression increased cellulose content, which is crucial for biofuel production. Our work shows that the activation tagging approach applied even on a non-genome saturation scale in a poplar tree can be successfully used for the discovery of genes positively modify biomass productivity. Such dominant forward genetics approaches can aid in biotechnological manipulation of woody biomass traits and help unravel the functions and mechanisms of individual genes, gene families, and regulatory modules.

## Introduction

Dedicated bioenergy crops like poplar, willow, and others are projected to displace 30 % of current US petroleum consumption (Perlack et al., 2005). In addition, woody biomass provides a renewable resource for production of pulp and paper, structural construction timber and multiple other products (de Vries et al., 2021; Skog, 2008). Despite the economic and ecological importance of woody biomass, the underlying molecular mechanisms of biomass-related traits remain poorly understood, and this significantly curtails the speed and efficiency of their improvement.

Biomass is a complex trait resulting from the integration of numerous processes, encompassing molecular, cellular, developmental, physiological, and metabolic levels (Carpita and McCann, 2020; Li et al., 2024; Zhu and Li, 2023). There has been substantial and long-standing interest in understanding biomass-related traits from both improvement and fundamental perspective (Groover, 2005; Li et al., 2024; Zhu and Li, 2023). However, dissecting these traits is challenging due to their complexity and the long generation cycle of trees, which makes traditional genetic, and mutagenesis approaches impractical (Busov et al., 2005a).

The value of forward genetics approaches involving insertional and other forms of mutagenesis is well established (Alonso et al., 2003). However, these methods are difficult to use in trees, largely because of their long generation cycles. Only dominant approaches like activation tagging and full-length overexpression (FOX) approaches are feasible because they can generate mutations in the first generation (Busov et al., 2005a; Rauschendorfer et al., 2020). Activation tagging uses a T-DNA vector with strong constitutive enhancer elements positioned near its left or right border. Insertion of the T-DNA into the genome typically leads to the up-regulation of a proximal flanking gene, resulting in a gain-of-function, dominant mutation (Deng et al., 2020). Dissecting gene functions through loss-of-function mutations is challenging because many genes exist in multiple copies, often organized in large gene families with partially redundant functions. Activation tagging, which creates gain-of-function mutations, offers an alternative for functional characterization of gene families (Deng et al., 2020; Nakazawa et al., 2003). The presence of such gene families is a particular problem in poplar, which has undergone whole-genome duplication events (Tuskan et al., 2006). Indeed, many of the genes we identified through activation tagging in *Populus* belong to large gene families such as Gibberellin (GA) 2-oxidase, AP2/ERF transcription factor (TF), AT-hook domain TF, and Lateral Organ Boundary (LBD) TF (Azeez et al., 2021; Busov et al., 2003; Trupiano et al., 2013; Yordanov et al., 2010, Yordanov et al., 2014). Activation tagging also offers several other advantages: easy characterization of the insertion site using the tag sequence (Liu et al., 1995); preferential insertion in gene-rich genomic regions (Kim and Veena, 2007); and discovery of poorly annotated or non-protein coding loci (Palatnik et al., 2003).

Here, we demonstrate the successful application of activation tagging in poplar to identify genes that affect biomass traits. Notably, we were able to identify a large number of mutations that positively affect one or several biomass-associated traits. For a subset of these mutants, we mapped the tag within the genome, identified the proximal genes, confirmed their upregulation, and recapitulated the mutant phenotypes via overexpression of the putative causative genes. Our findings demonstrate the efficacy of a dominant forward tagging approach applied on a non-genome saturation scale for uncovering genes that positively impact biomass traits in a tree.

## Results

### Phenotypic screens for mutations affecting biomass-associated traits

**Figure 1:**
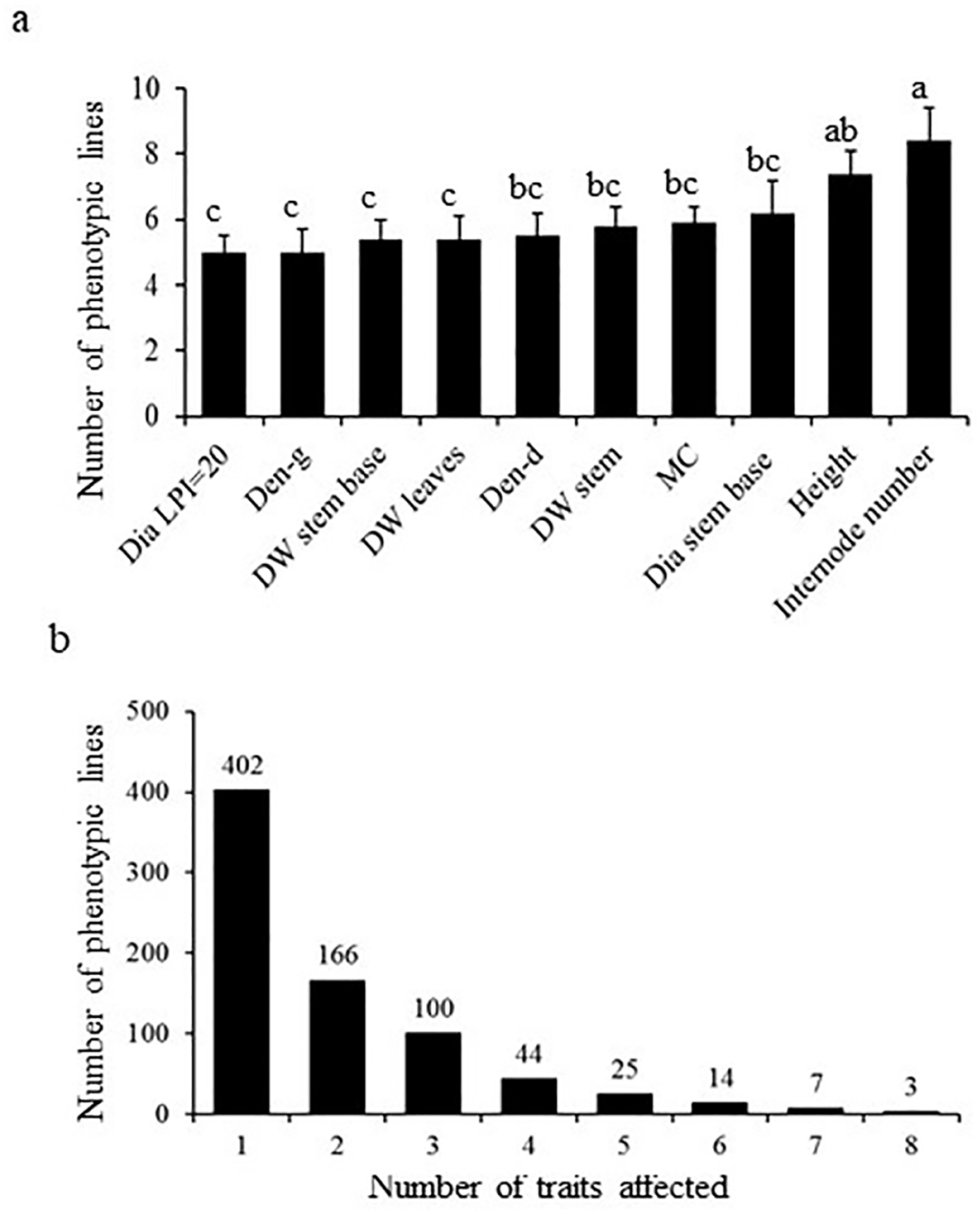
Summary of mutants and biomass traits affected in the poplar activation tagging screen. **(A)** Number of mutants identified to be positively and significantly affected in the various measured biomass-associated traits. Different letters indicate significance as determined by a one-way ANOVA followed by Fisher’s test (*p* < 0.05). **(B)** Illustrate the number of mutants affected in single versus multiple biomass traits.

To identify genes influencing traits linked to biomass growth in *Populus*, we generated and screened under controlled greenhouse conditions a population of 2,700 activation-tagged lines (see Material and Methods). We focused on traits that are linked to biomass growth (**Figure 1A**). A total of 761 lines exhibited significant effect on at least one trait, categorized as phenotypic mutant (**Figure 1B**). The various mutations had an approximately evenly distributed impact over the 10 studied traits. The number of internodes showing the greatest impact among all traits (**Figure 1A**). In contrast, traits such as diameter at leaf plastochron index (LPI) 20, green density, dry weight at the base and dry leaf weight were least affected (**Figure 1A**). More than half of the mutants (402) were affected in only one trait (**Figure 1B**). In many cases, mutations impacted simultaneously as many as 5-8 traits (**Figure 1B**).

### Correlations between affected phenotypic traits

To explore potential correlation between traits, pair-wise phenotypic correlations were calculated across the entire experiment (**Table 1**). As expected, strong correlations were evident between closely related traits, such as green and dry density, and between total dry biomass of the whole stem and the stem base. Interestingly, less intuitive correlations were also observed. For instance, dry leaf biomass showed significant and positive correlation with the stem base diameter and dry stem biomass. Additionally, dry stem biomass correlated positively with internode number and the stem base diameter. These correlations may suggest underlaying morphological or physiological connections that are not apparent and require further investigations.

**Table 1:** Correlation between different phenotypic traits.

	Height	Int #	Dia stb	Dia LPI20	DW stb	MC	Den-g	Den-d	DW st	DW leaves
**Height**	1									
**Int #**	0.40	1								
**Dia stb**	0.50*	0.60**	1							
**Dia LPI20**	-0.17	0.09	0.15	1						
**DW stb**	0.19	0.35	0.56**	0.28	1					
**MC**	0.12	0.14	0.23	0.13	0.33	1				
**Den-g**	0.01	0.34	0.20	0.28	0.34	0.55**	1			
**Den-d**	0.08	0.25	0.24	0.12	0.32	0.55**	0.86***	1		
**DW st**	0.38	0.54**	0.61***	0.19	0.70***	0.32	0.41	0.37	1	
**DW leaves**	0.14	0.41	0.59**	0.16	0.58**	0.31	0.17	0.26	0.51**	1

Regression analysis was used to test the significance in the correlation between pair-wise traits. “*”, “**” and “***” marked in bold denote significant correlation at p ≤ 0.05, p ≤ 0.01 and p ≤ 0.001, respectively. int # - internode number, dia – diameter, stb - stem base, st – stem, LPI20 – leaf plastochron index 20, dw – dry weight, mc – moisture content, den-g – density green, den-d – density dry.

### The tag insertions are proximal to genes

**Figure 2:**
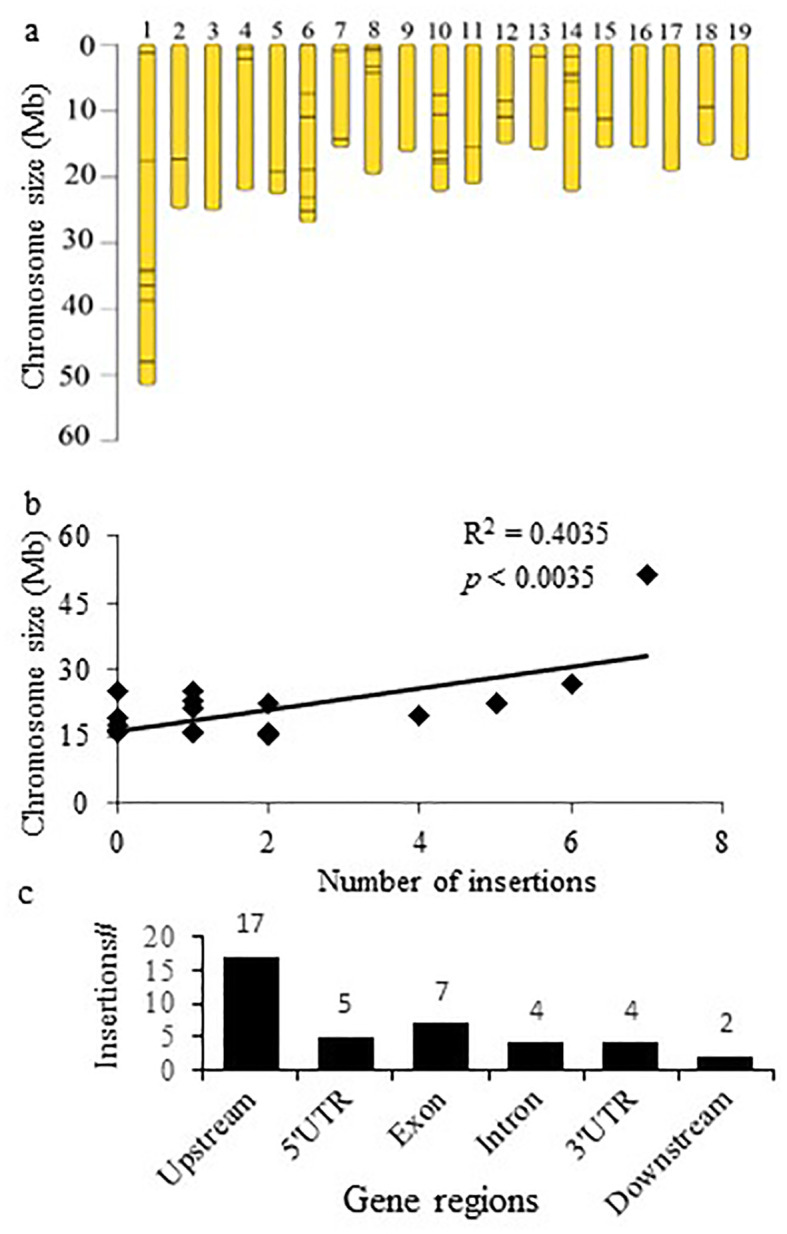
Characterization of the activation tag integration in the *Populus* genome. **(A)** Distribution of the insertions within the chromosomes. **(B)** Correlation between chromosome size and number of insertions per chromosome. **(C)** Predominant identification of T-DNA insertions upstream from the gene coding region.

We characterized T-DNA insertions (**Supplementary Table S1**) in 40 mutant lines (**Table 2**). Chromosome 1 had the highest number of insertions (7), followed by 6 insertions on chromosome 6, and 5 insertions on each chromosome 10 and 14 (**Figure 2A**). A significant correlation between chromosome size and the number of insertions suggests random insertion of the tag (**Figure 2B**). Most (37.5%/15) of the insertions were located within the 10 Kbp 5′/3′ regions proximal to genes, while 17.5 % (7) in exons and 12.5 % (5) in introns (**Figure 2C**). Additionally, 22.5 % (9) were positioned in the 5’- or 3’-untranslated gene regions (UTR) (**Figure 2C**).

**Table 2:** Growth and biomass yield are significantly increased in poplar activation tagging lines.

Line ID	Trait significantly affected			
	Total #	* (*p* < 0.05)	** (*p* < 0.01)	*** (*p* < 0.001)
A634-2	8	Internode #; DW stem base, stem	Height; Dia stem base; MC;	Den-d
			Den-g	
826L-3	8	Height; Internode #; Dia stem base;	DW leaves	MC
		DW stem base; Den-d; DW stem		
A771-3	6	MC	Dia stem base; Den-d; DW stem base, stem, leaves	n.a.
A885-1	6	Height; DW stem base; Den-g	Den-d; DW stem	MC
A943-1	6	Dia LPI20; DW stem, leaves	Dia stem base; DW stem base; MC	n.a.
A630-7	5	DW stem base, stem, leaves;	Height; Internode #	n.a.
A726-3	5	Internode #; DW stem	Height; Dia stem base	n.a.
A857-2	5	Dia LPI20	Internode #; DW stem	Height; DW leaves
A934-2	5	Height; MC; DW stem, leaves	n.a.	Internode #
B21-1	5	MC; DW stem	Den-d	DW stem base; Den-g
B0-4	5	Height; Internode #; Dia LPI20;	n.a.	Den-g
		DW stem		
795L-6	5	Height; Internode #; Dia base;	n.a.	n.a.
		DW stem base; Den-d		
A975-4	5	Height; Dia stem base; Den-d; DW stem	n.a	Den-g
A689-4	4	Dia LPI20; DW stem, leaves	Dia stem base	n.a.
A835-3	4	Internode #; Dia stem base; MC	Den-d	n.a.
A927-3	4	MC; Den-g; Den-d; DW leaves	n.a.	n.a.
A635-1	4	Dia stem base; Den-g	n.a.	DW stem base; Den-d
A822-3	4	Height; Internode #; DW stem base	n.a.	DW stem
A863-3	4	MC; Den-g	DW stem base; Den-d	n.a.
A836-1	4	DW leaves	DW stem base, stem	Internode #
A955-2	4	Dia stem base; DW stem	DW stem base	MC
A991-1	4	Den-g; Den-d; DW stem, leaves	n.a.	n.a.
501L-5	4	Height; Dia stem base; DW stem base, DW stem	n.a.	n.a.
A541-1	3	Dia stem base, LPI20	n.a.	Internode #
A826-3	3	Height; DW stem, leaves	n.a.	n.a.
345L-1	3	DW stem base; Den-d	Dia stem base	n.a.
A613-2	3	MC	Den-g; Den-d	n.a.
575L-1	3	Den-d	n.a.	Height; Internode #
A979-4	3	MC; DW leaves	Dia LPI20	n.a.
707L-1	3	MC; Den-d	Den-g	n.a.
994L-1	3	MC; Den-d	n.a.	Den-g
659L-1	3	Internode #s; Den-d	n.a.	Den-g
A901-5	2	n.a.	n.a.	Dia LPI20; DW stem
A842-3	2	Dia stem base	DW leaves	n.a.
A82-2	2	Height; Dia stem base	n.a.	n.a.
A862-1	2	Dia stem base; Den-d	n.a.	n.a.
199p-5	2	Internode #; Dia LPI20	n.a.	n.a.
A915-2	2	n.a.	n.a.	Internode #; DW stem
A874-9	2	n.a.	n.a.	Internode #; Den-g
A955-1	1	Dia LPI20	n.a.	n.a.

Various phenotypic traits were positively impacted in the lines selected for the analysis of the insertion of the tag. Within the subset each individual line was compared to the entire subset by trait and significant differences were determined by Student’s t-test (“*”, “**” and “***” denoting p < 0.05, p < 0.01 and p < 0.001, respectively). n.a. – not available, Total # ‘Total trait(s) number’.

### Tagged genes are activated

For nine mutants, we proceeded with further characterization of the expression of the genes near to the insertion sites (**Table 3**). These mutants were selected based on the magnitude of their impact on the affected trait, the number of traits affected, and the novelty of the gene function (**Table 3**). Since the tag is often inserted near multiple genes, and thus potentially impacts more than one, we studied the expression of all potential candidates. In most cases (8 out of 9 lines), we found only one of the nearby genes activated (**Table 3**). However, in only one line (A630-7), both genes proximal to the tag showed activation.

**Table 3:** Real-time quantitative RT-PCR analysis of proximal genes flanking the T-DNA insertion site.

Genotype	Relative expression	
	Left flanking gene	Right flanking gene
	*PtXaTreH.14G052500*	*PtXaTreH.14G052600*
WT-717	84.02 ± 3.9	0.01 ± 0.0
A630-7	271.27 ± 22.9*	0.83 ± 0.1*
	*PtXaAlbH.08G010800*	*PtXaAlbH.08G010900*
WT-717	0.15 ± 0.01	n.d.
A771-3	0.71 ± 0.00**	n.d.
	*PtXaAlbH.05G161200*	*PtXaAlbH.05G161300*
WT-717	0.05 ± 0.01	0.03 ± 0.02
A541-1	0.46 ± 0.02*	0.02 ± 0.01
	*PtXaAlbH.10G146600*	*PtXaAlbH.10G146700*
WT-717	1.44 ± 0.10	0.12 ± 0.00
A689-4	0.24 ± 0.01	2.28 ± 0.14**
	*PtXaAlbH.10G086100*	*PtXaAlbH.10G086200*
WT-717	43.61 ± 3.2	n.d.
A842-3	51.70 ± 2.2	499.36 ± 18.6***
	*PtXaTreH.15G090100*	*PtXaTreH.15G090200*
WT-717	0.62 ± 0.04	0.03 ± 0.0
A835-3	0.71 ± 0.03	0.06 ± 0.0*
	*PtXaTreH.14G114800*	*PtXaTreH.14G114900*
WT-717	0.03 ± 0.001	n.d.
A726-3	0.20 ± 0.001***	n.d.
	*PtXaTreH.01G014500*	*PtXaTreH.01G014600*
WT-717	12.06 ± 0.8	n.d.
A826-3	42.96 ± 4.9*	n.d.
	*PtXaTreH.06G072700*	*PtXaTreH.06G072800*
WT-717	7.72 ± 0.2	0.55 ± 0.01
A927-3	12.85 ± 0.9*	0.65 ± 0.05

Values represent relative expression (mean ± standard error) of three biological replicates. Ubiquitin (Ubq) was amplified as a normalization control. Asterisks “*”, “**” and “***” indicate significance compared to the wild type as determined by Student’s t-test at p < 0.05, p < 0.01 and p < 0.001, respectively. n.d. – not detected.

### Tagged genes show diverse and tissue-specific native expression patterns

**Figure 3:**
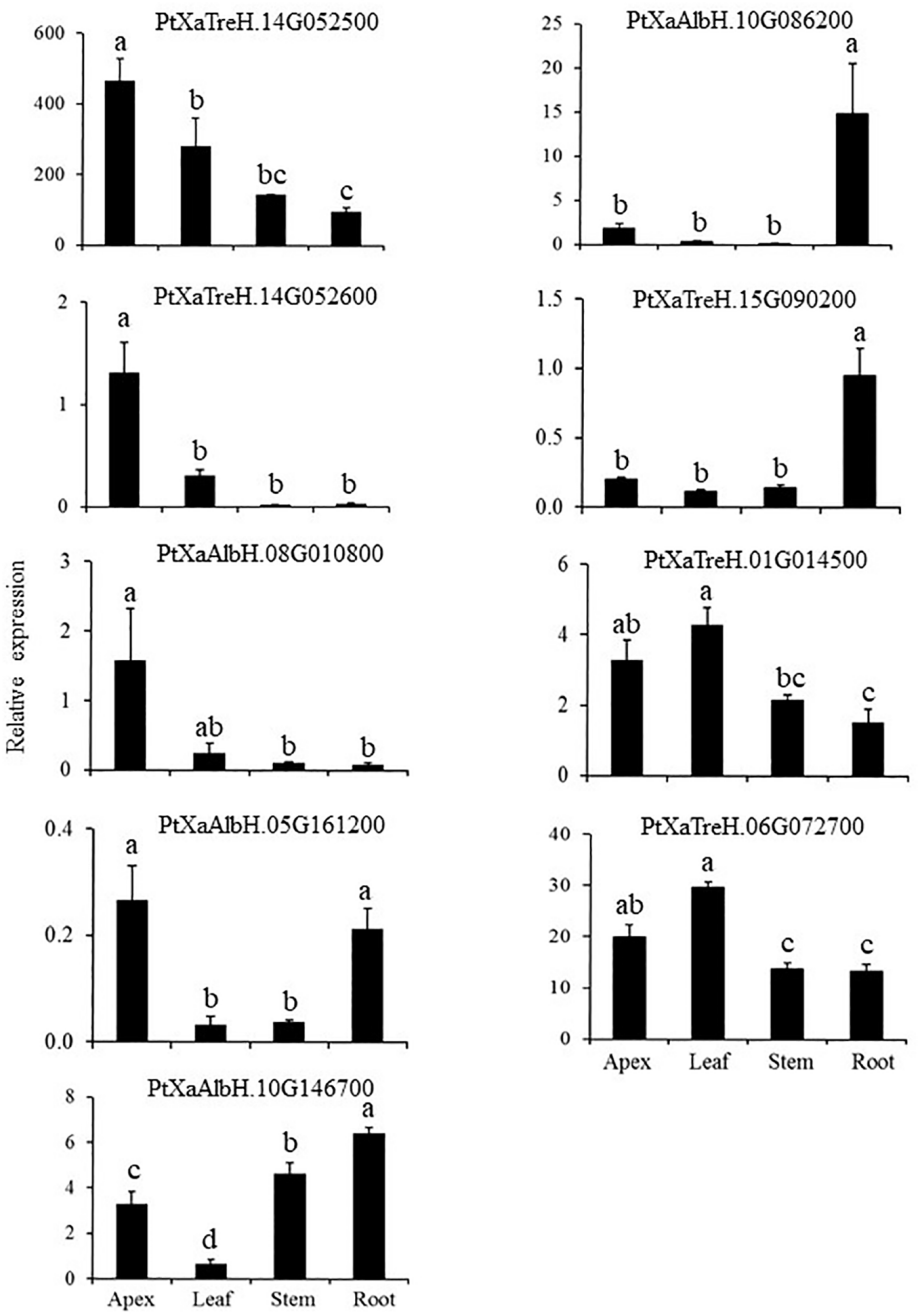
Expression of the tagged genes in different organs/tissues. Bars represent means of four biological replicates ± standard error. Different letters indicate significant differences as determined by a one-way ANOVA followed by Fisher’s test (*p* < 0.05).

We characterized the expression of the activated genes in various organs, including the apex, leaf, stem, and root of wild type (WT-717) plants (**Figure 3**). These genes exhibited diverse tissue specific expression patterns, suggesting varied influence on biomass traits. For example, *PtXaTreH.14G052500*, *PtXaTreH.14G052600* and *PtXaAlbH.08G010800* were predominantly expressed in the apex. *PtXaAlbH.10G086200* and *PtXaTreH.15G090200* were abundant in the root, whereas *PtXaAlbH.05G161200* was mainly expressed in both the apex and roots. *PtXaTreH.06G072700* was expressed in all studied tissues.

### Fasciclin-like gene enhances multiple biomass traits

One of the lines selected for recapitulation experiments was A630-7, chosen for its simultaneous impacts on multiple traits (five in total, as shown in **Table 2**). Most importantly, the mutation significantly enhanced stem dry weight, a crucial aspect of biomass production. Additionally, the line exhibited increased leaf dry biomass, which, as previously mentioned, is positively correlated with stem dry weight (**Table 1**). This prompted our interest in identifying the gene responsible for these changes. Position and expression analyses indicated the upregulation of two genes near the tag insertion site (**Table 3**). One gene showed the highest homology to *Arabidopsis's Fasciclin 10* (*FLA10*), which we named *PtaFLA10*. The other gene exhibited the highest sequence homology to *Growth Regulating Factor 9* (*GRF9*) transcription factor from *Arabidopsis*, which was called *PtaGRF9*. It was unclear which of the two genes was responsible for the phenotypic changes. We thus produced overexpression constructs for both genes and transformed them into transgenic plants. Numerous independent events were regenerated with the *PtaFLA* overexpression construct (*oe-PtaFLA10*). However, despite several transformations attempts, we could only recover four transgenic plants with *PtaGRF9* overexpression construct (*oe-PtaGRF9*), suggesting that *PtaGRF* interferes with the regeneration process.

**Figure 4:**
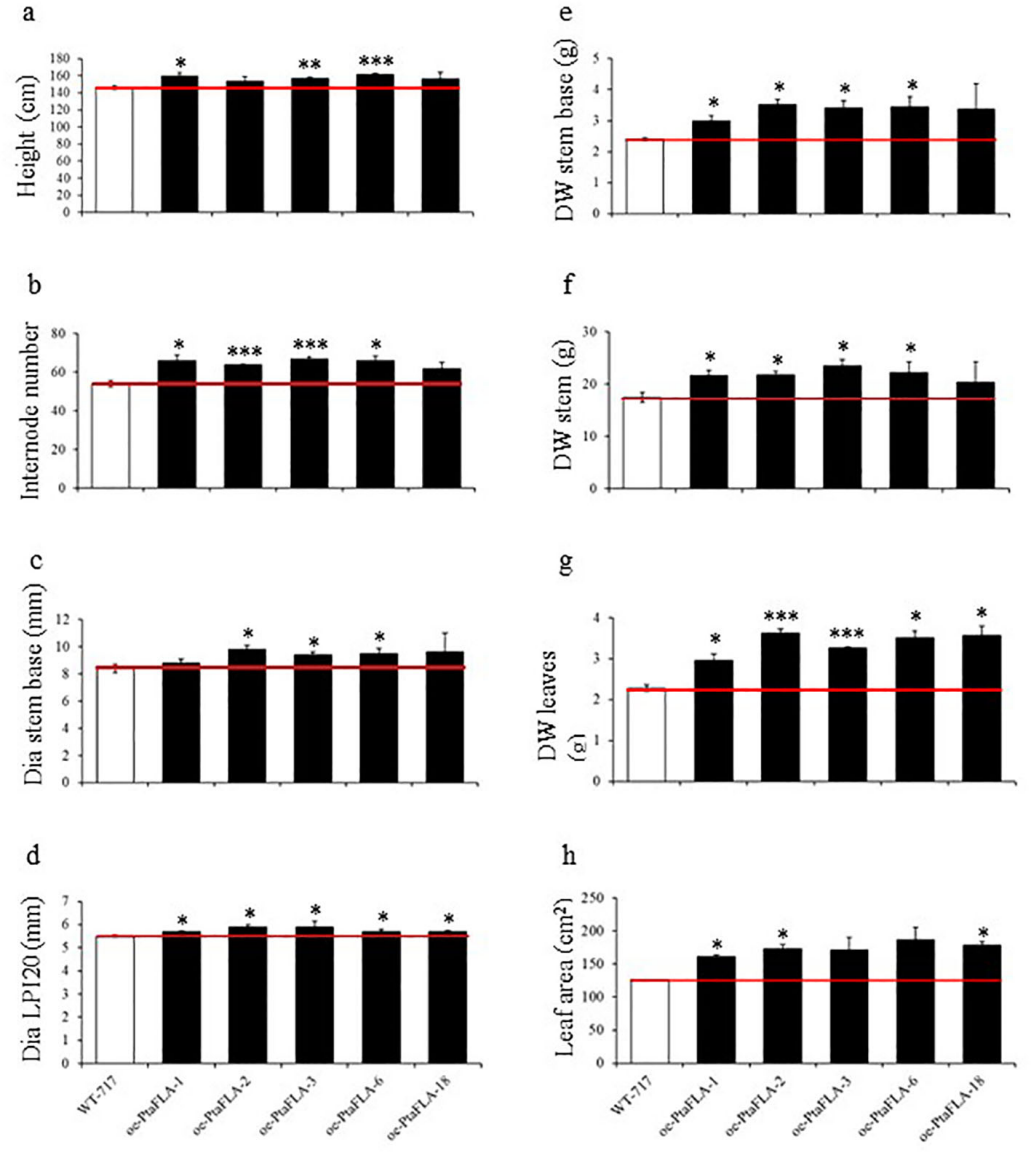
Recapitulation of A630-7 phenotype. **(A–H)** Changes of traits in mutant and *oe*-*PtaFLA* lines. Values are reported as mean ± SE (*n* = 4). White bars represent wild type and black bars – *PtaFLA* OE=overexpression lines. Asterisks indicate significant differences between transgenics and wild type plants as determined by Student’s *t*-test (*, ** and *** denoting *p* < 0.05, *p* < 0.01 and *p* < 0.001, respectively). Red line corresponds to the wild types’ threshold.

*PtaFLA10* overexpression positively affected several phenotypic traits linked to biomass growth compared to WT-717 plants (**Figure 4**). For instance, both the original mutant A630-7 line and *oe-PtaFLA* lines were about 9 % taller than the wild type. Additionally, the number of internodes increased by 15% in the A630-7, while the *oe-PtaFLA* lines exhibited an average increase of 20 % (**Figure 4**). The *oe-PtaFLA* lines also displayed changes not observed in the original mutant, likely due to the strong overexpression. For example, there was a significant increase in the diameter at the 20^th^ internode and at the stem base (**Figure 4**). Most notably, a 40 % increase in the dry stem biomass was observed in both the original A630-7 mutant line and *oe-PtaFLA* transgenics. Consistent with the strong correlation between leaf and stem dry weight (**Table 1**), leaf dry weight was also increased in both the mutant and recapitulation transgenics.

In addition to enhanced biomass growth, both the original line and the overexpression transgenics showed a significant decrease in lignin content (**Table 4**). The overexpression transgenics also exhibited a reduced S/G ratio, a change not observed on original line (**Table 4**). No significant changes were measured in cell wall carbohydrates.

**Table 4:** Cell wall characteristics are altered in the mutants and recapitulated lines.

Genotype	Lignin (µg mg^-1^ DW)	S/G ratio	Hemicellulose (C5) (µg mg^-1^ DW)	Cellulose (C6) (µg mg^-1^ DW)
WT-717	238.44 ± 2.6	1.55 ± 0.04	280.36 ± 1.5	333.23 ± 4.7
A630-7	225.12 ± 2.9*	1.58 ± 0.01	282.40 ± 0.8	344.95 ± 0.2
*oe-PtaFLA*	229.59 ± 0.9*	1.44 ± 0.02*	277.38 ± 3.5	341.88 ± 3.6
WT-717	232.82 ± 1.4	1.55 ± 0.05	266.13 ± 3.1	318.14 ± 2.5
A541-1	238.77 ± 3.4	1.65 ± 0.05	271.20 ± 3.6	317.08 ± 1.2
*oe-PtaPAT*	243.75 ± 1.4***	1.41 ± 0.01*	265.84 ± 2.3	327.64 ± 2.0*

Changes in the lignin, S/G ratio, hemicellulose and cellulose content were determined by using PyMBMS analysis. Values are presented as mean ± SE (*n* = 4). For OE lines the data are presented as mean of at least five individual lines. Asterisks indicate significant differences from the wild type (WT-717) as determined by Student’s *t*-test (* and *** denoting *p* < 0.05 and *p* < 0.001, respectively). Significant changes are highlighted in bold.

Overexpression of the other gene, *PtaGRF9*, did not result in significant phenotypic changes, suggesting that the observed mutant phenotypic characteristics are due to the upregulation of *PtaFLA10* gene.

### A patatin-like gene affects biomass growth, leaf development and response to drought

**Figure 5:**
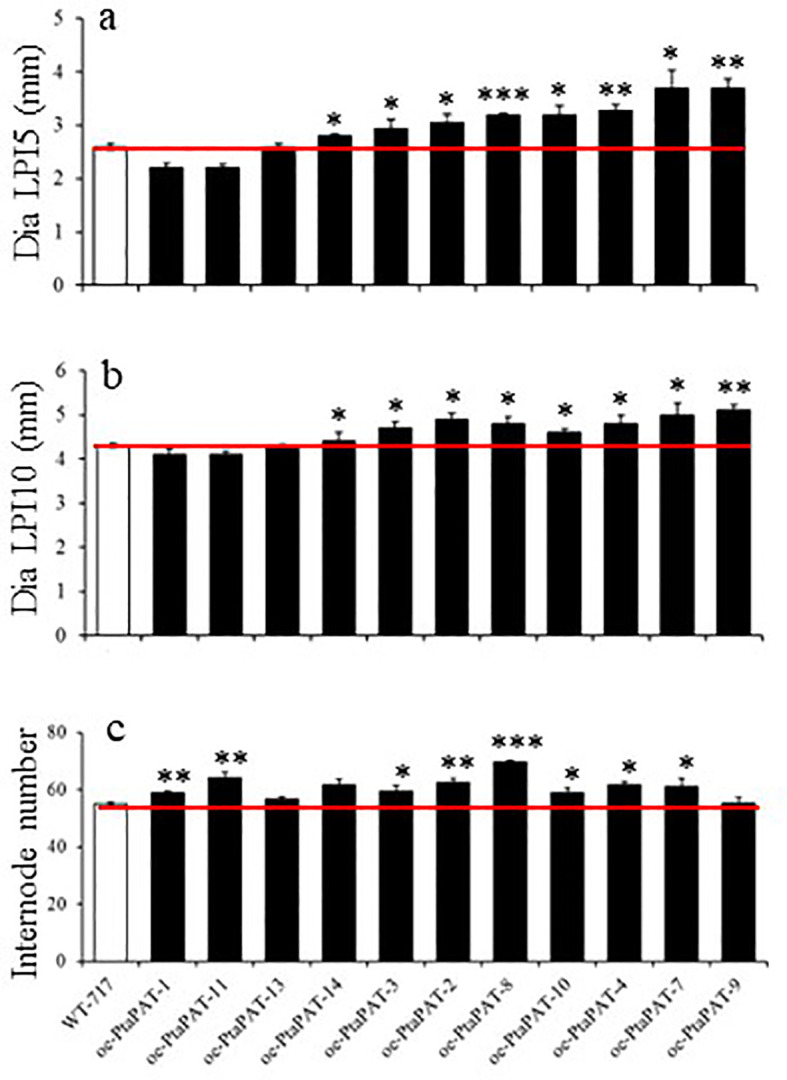
Recapitulation of A541-1 phenotype. **(A–C)** Changes in traits of oe-PtaPAT lines. Bars represent mean of four biological replicates ± SE (n = 4). White bars represent wild type and black bars represent Patatin OE lines. Asterisks indicate significant differences between transgenic and WT plants as determined by Student’s t-test (‘*’, ‘**’ and ‘***’ denoting p < 0.05, p < 0.01 and p < 0.001, respectively).

**Figure 6:**
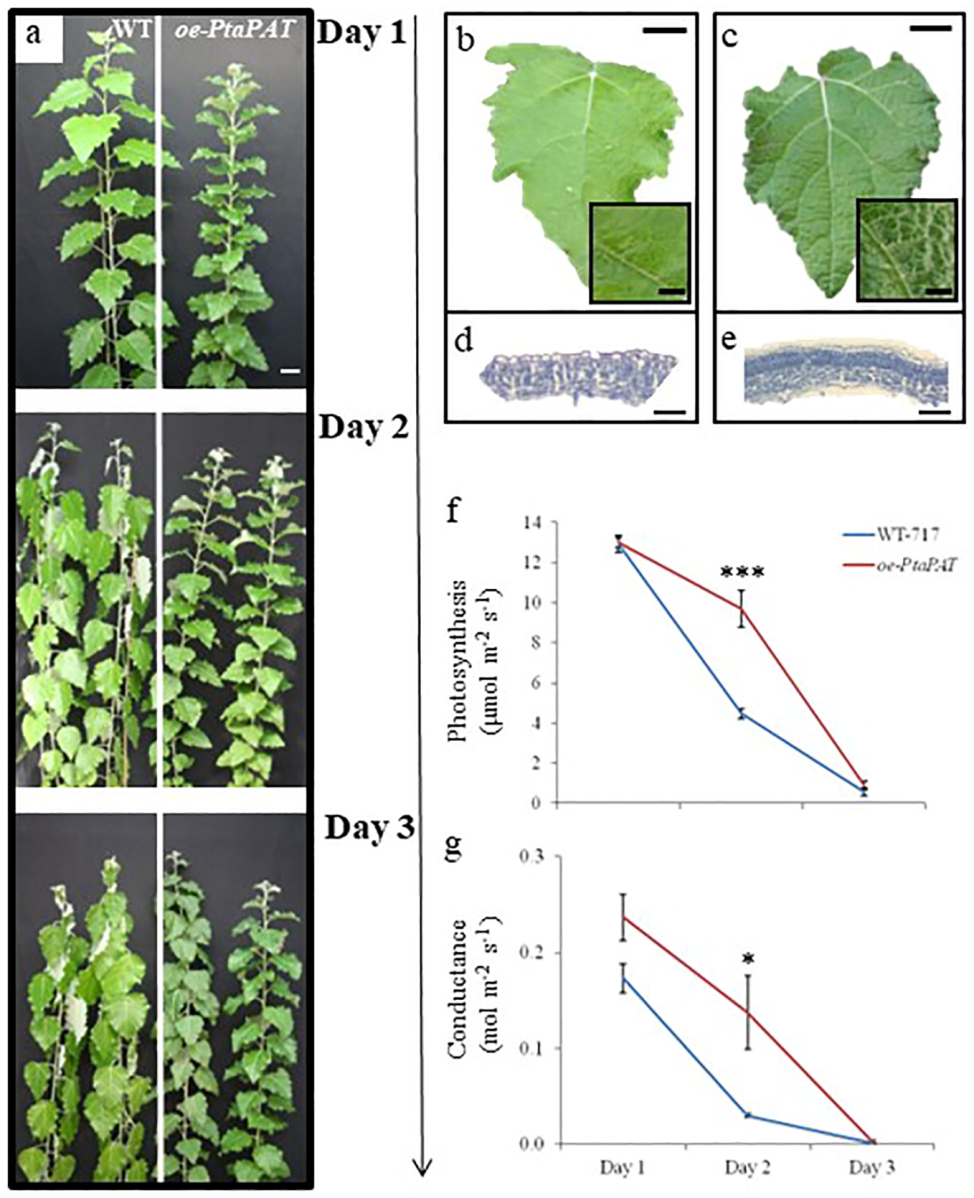
Poplar patatin OE lines showed better performance under drought conditions. **(A)** Drought experiment including wild type (WT-717) and *oe-PtaPAT* lines. Plants were maintained without water for 3 days. Pictures were taken daily and show representative appearance of multiple ramets and lines. Leaf texture and cross-section of WT-717 **(B, D)** and *oe-PtaPAT* **(C, E)** control plants (n=3 lines with 4 ramets per line). Analysis of photosynthetic activity and **(F)** stomatal conductance **(G)** during drought stress. Data were collected every day during the treatment period. Asterisks indicate significant differences between transgenic and WT-717 plants as determined by Student’s *t*-test (n=4, * and *** denoting *p*< 0.05 and *p* < 0.001, respectively). Scale bars **(A)** = 5 cm, **(B, C)** = 2 cm, **(D, E)** = 50 µm.

We also further characterized the A541-1 mutant line through recapitulation experiments. The activated gene in this line encoded a protein with high similarity to patatin, and thus named PtaPAT. *PtaPAT* overexpression led to an increase in stem diameter, but only in stems undergoing primary growth, specifically 5^th^ and 10^th^ internodes (**Figures 5A, B**; **Table 2**). Consistent with the original A541-1 mutant line, the *oe-PtaPAT* lines also exhibited an increased number of internodes (**Figure 5C**; **Table 2**). Additionally, overexpression of PtaPAT resulted in leaves with an uneven adaxial leaf surface, reminiscent of potato leaves (**Figures 6A–C**).

Given the distinct leaf surface observed in the *oe-PtaPAT* lines, we performed histological analysis (**Figures 6 D, E**). The *oe-PtaPAT* lines displayed a much denser palisade mesophyll cell layer, with a larger number of closely spaced cells compared to the WT plants (**Figures 6 D, E**).

Because of these unique changes in leaf structure, we wanted to study the performance of the *oe-PtaPAT* lines under drought conditions (**Figures 6A, F, G**). Wild-type and *oe-PtaPAT* lines were subjected to drought stress for 3 days and CO_2_ assimilation and stomatal conductance were monitored during this period. On the 2^nd^ day of the stress treatment *oe-PtaPAT* lines were not wilted (**Figure 6A**) and showed higher photosynthetic activity (**Figure 6F)** and stomatal conductance (**Figure 6G**) compared to the wild-type plants. These results indicate a possible and yet unknown role of the patatin gene in the response to the drought and create new opportunities for further gene manipulations to improve drought tolerance in plants.

The *oe-PtaPAT* lines also showed increases in lignin, cellulose, and S/G ratio, changes not observed in the original mutant line and likely attributable to the much higher level of overexpression (**Table 4**).

## Discussion

In this study, we applied a moderate, non-genome saturation size of poplar activation tagging population to discover genes that positively affect biomass. Despite the moderate population size and apparent randomness of the tag insertion, we identified a significant number of ‘productive’ mutations. Several pieces of evidence suggest that these mutations result from genuine activation tagging. These include the tag’s insertion near genes, the upregulation of the adjacent genes, and the successful recapitulation of the phenotypic changes through overexpression. The high success of activation tagging in poplar is attributed to the preferential insertion of the T-DNA near genes, likely due to Agrobacterium's tendency to target transcriptionally active, open chromatin regions, a phenomenon also observed in Arabidopsis and rice (Alonso et al., 2003; An et al., 2003; Chen et al., 2003; Forsbach et al., 2003; Rosso et al., 2003; Sallaud et al., 2004; Szabados et al., 2002). The bias toward open chromatin enhances the likelihood of successful activation and productive mutation, while our analysis revealed a random insertion pattern correlated with chromosomal size. Majority (37.5%) of the positioned tags were found with the 10 kb upstream or downstream of the genes, confirming our previous finding (Busov et al., 2010) with a much smaller population. Finally, the large size of poplar also facilitates the detection of the phenotypic changes. As a perennial species, these phenotypic changes could be further accentuated if the screening were performed under multi-year field conditions, where the changes would accumulate over time. Unfortunately, due to stringent regulatory regimes, conducting such field trials remains logistically challenging.

There is substantial evidence indicating significant interdependence between the regulators of biomass production and cell wall thickening (Maleki et al., 2020). Similar findings were observed in *Arabidopsis* (Hu et al., 2018). For instances, enzymes involved in cell wall loosening result significant increases in biomass accumulation (Park et al., 2003; Shani et al., 2004), highlighting the intricate link between cell wall biology and growth processes in plants. Overexpression of a cellulose synthase led to increased biomass production in poplar (Maleki et al., 2020). Therefore, it is not surprising that both genes we recapitulated (e.g., patatin and fasciclin) are associated with cell wall metabolism.

Fasciclins have long been known to be involved in secondary wall thickening, though their exact role remains elusive (Dharmawardhana et al., 2010; Huang et al., 2013; Janz et al., 2012; Lafarguette et al., 2004; Ma et al., 2022, Ma et al., 2023; MacMillan et al., 2010; Wang et al., 2015a, Wang et al., 2017, Wang et al., 2015a). Fasciclin-like arabinogalactan proteins (FLAs) contain a characteristic fasciclin-like domain, which plays a crucial role in cell-cell and cell-matrix interactions, as well as in cell expansion, in animal cells. Recent studies have identified approximately 50 FLA proteins in *Populus trichocarpa* (poplar), with most of these proteins being highly expressed in developing xylem. Notably, group A FLAs are specifically associated with lignified internodes, highlighting their potential role in wood formation and structural integrity (Zhen et al., 2023). Indeed, the *PtrFLA40/45* mutant exhibited a significant increase in lignin content, which was accompanied by the upregulation of six lignin biosynthetic genes (Zhen et al., 2023). Interestingly, several other FLA genes in poplar have been implicated in the formation of tension wood, operating through a pathway associated with Gibberellin A3 signaling (Wang et al., 2017; Lafarguette et al., 2004). Since secondary cell wall constitutes the bulk of lignocellulosic biomass, it is not surprising that the modification in fasciclin expression leads to changes in biomass yield and properties. The fasciclin family is large and complex with a significant variation in the number and types of domains (Dharmawardhana et al., 2010). The gene identified in our study is of particular interest for several reasons, First, it increases biomass by 40 % on a dry biomass basis. Additionally, the cell wall shows a significant decrease in lignin content. These changes are highly consistent with the effects observed with other members of the fasciclin family (Ma et al., 2022, Ma et al., 2023; Wang et al., 2015a).

Patatins, a nonspecific lipid acyl hydrolase reported to play a role in plant signaling were only recently linked to cell wall metabolism, specifically lignin and cellulose biosynthesis (Huang et al., 2001; Jang and Lee, 2020; Li et al., 2011; Simiyu et al., 2023). These lipolytic enzymes, primarily known for their role in lipid metabolism, have an yet unclear link to lignin biosynthesis. However, substantial experimental evidence, including in poplar (Jang and Lee, 2020), is pointing to a connection between lipid metabolism and lignin biosynthesis pathway (Huang et al., 2001; Jang and Lee, 2020; Li et al., 2011; Simiyu et al., 2023). One compelling explanation presented by Ali et al. is that patatins play a pivotal role in regulating central carbon flux during cell wall biosynthesis (Ali et al., 2022). Interestingly, one of the first patatins identified was through activation tagging in *Arabidopsis* (Huang et al., 2001). The patatin gene was named *STURDY*, after the mutant’s tougher stems, resulting from changes in lignin biosynthesis (Huang et al., 2001).The mutant also displayed increased stem girth due to enhanced cell proliferation.

Additionally, our study uncovered a fascinating link between patatins and drought stress response, which was not observed in the original mutant but only when the gene was highly overexpressed. It is unclear if this is a pleiotropic effect of the ectopic expression or a result of the much higher expression levels than in the WT plants. Nevertheless, the evidence is pointing to a highly positive effect of patatin overexpression on drought resistance. We are still unclear of the underpinnings of this phenotype. One potential factor is the denser leaf structure, which may reduce transpiration levels, as suggested by our measurements of photosynthesis and stomatal conductance. Given that the patatins are lipolytic enzymes, their role in drought resistance might involve a wide array of changes, such as membrane organization, mobilization of storage reserves, and modification of the cell’s osmotic potential (Li et al., 2020; Scherer et al., 2010). Patatins are also induced by abiotic stress, suggesting they likely play a significant yet unknown role in stress responses (Li et al., 2020; Matos et al., 2001, Matos et al., 2008). Further investigations into this underlying mechanism could lead to new strategies for engineering stress response. Most excitingly, our work indicates a simultaneous increase in biomass yield and drought resistance, unlike many other strategies for engineering drought resistance that often result in growth penalties (Hwang et al., 2010; Schluepmann et al., 2012; Sreenivasulu et al., 2012).

Fascinatingly, the leaf surface of the patatin overexpression transgenics resembles the leaf morphology of potato leaves, where patatin is highly expressed, particularly in the tubers (Andrews et al., 1988; Rosahl et al., 1986). The change in leaf morphology and tissue organization are reminiscent of the mutant phenotypes associated with disruption in hormone metabolism and/or signaling, leading to imbalances in cell division, proliferation and differentiation. Some studies linked patatins to auxin, which could explain the significant growth/developmental phenotypes observed in our study (Dong et al., 2014; Labusch et al., 2013).

Our work demonstrates the feasibility and efficacy of activation tagging for discovering genes that positively influence biomass-associated traits in poplar. Recapitulation experiments indicate that greater improvements can be achieved through overexpression, with additional enhancements such as drought resistance identified, suggesting potential for simultaneously improving plant growth and resilience.

## Methods

### Plant transformation and validation

A hybrid aspen clone, *Populus tremula x Populus alba* INRA 717-IB4 (referred to as WT or 717), was used in all experiments, including the transgenic manipulations. The activation tagging population was generated using a binary vector pSKI074 via an *Agrobacterium*-mediated procedure (Han et al., 2000). All putative transformants were PCR verified for the presence of neomycin phosphotransferase II (NPT), as a selectable marker (Yordanov et al., 2010). Only the NPT-positive transformant were used in further experiments. These verified transformant were propagated and maintained *in vitro* on ½ MS media with 20 g/l sucrose (Caisson), 0.1mg/l IBA (Sigma-Aldrich), vitamins (Han et al., 2000) solidified with 2.5 g/l Gelrite (Sigma) and 4 g/l Phytablend agar (Caisson), at 16/8 h day/night photoperiod (20 µmol m^-2^s^-1^).

### Plant growth conditions

For the greenhouse experiments, plants were first propagated and grown *in vitro* for four weeks on ½ MS solid media (as describe above). The rooted plantlets were then transferred to soil and gradually acclimated to greenhouse conditions. Once acclimated, uniformly developed plants were transplanted and grown in greenhouse for approximately 3 months as previously described (Rauschendorfer et al., 2020). The experiment was conducted in a completely randomized design with three replications.

### Biometric measurements and harvesting

Measurements of the height and basal diameter of the stem, counting of the internodes of each plant were performed regularly to analyze the growth characteristics of poplar plants. A slide digital caliper was used to determine the basal diameter of each plant above the pot surface. For harvest the above-ground part of each plant was separated into leaves and stems. The fifth leaf from the top of the plant was used to determine the leaf area. Digital images were taken with Nicon Coolpix camera. Leaves and stems were air dried, and their dry weight was measured until was unchanged. We measured green wood density (Den-g, g/cm^3^), basic/dry wood density (Den-d, g/cm^3^), and moisture content (MC, %) of wood samples collected at the base (15 cm from the soil surface) of the stem. For each sample, we determine the green disc mass and green volume, using water displacement. The stem sections were kiln-dried at 105°C and again weighed. Den-g and Den-d were estimated for each tree as sample mass (g) / disc green volume (cm^3^).MC will be estimated as [(Den-g – Den-d) / Den-g] x 100.

### Measurements of photosynthesis

Two-month-old greenhouse-grown plants subjected to drought stress, were used to measure photosynthesis. Net photosynthetic rate was measured using LI-6400XT portable photosynthesis system (Li-Cor Inc., Lincoln, NE, USA). The measurements were conducted during mid- and late- morning (usually 09:00 – 11:30am) on uniformly sunny days. Leaves of each genotype (four biological replicates/genotype) were measured under the following conditions: 400 μmol s ^-1^ flow rate, 400 μmol mol ^-1^ reference CO _2_ concentration and photosynthetic photon flux density (PPFD) of 1500 μmol m ^-2^ s ^-1^, provided by a red–blue light source (6400-02B). Relative humidity was maintained between 50 % and 75 % inside the chamber (RH_S_%). The control temperature was set at 30°C.

### Tag mapping and validation of gene activation

Recovery of sequence flanking the insertion site of the activation tag was performed as previously described (Yordanov et al., 2010). The isolated DNA fragments were positioned in the poplar 717 genome using BLAST searches in the Phythozome v13 (http://www.phythozome.net/poplar.php) database and proximal genes to the insertion site identified. Expression of the flanking genes was studied using RT-PCR with gene specific primers (**Supplementary Table S2**) and *Ubiquitin* (*Ubq*) gene (Wei et al., 2013) as a loading control.

### Generation of constructs and transformation

The open reading frame of candidate genes were amplified using the following primers (**Supplementary Table S3**). The amplified product was then cloned into pDONR221 vector (ThermoFisher Scientific) using the BP Gateway system (Invitrogen), sequence validated and transferred into the overexpression pK7WG2 vector (Karimi et al., 2002) using the LR Gateway system (Invitrogen). The binary vectors were transformed into Agrobacterium strain AGL1 (Lazo et al., 1991) and transformed into the hybrid aspen clone, *Populus tremula x Populus alba* INRA 717-IB4 as previously described (Han et al., 2000).

### Real time RT-PCR

Total RNA was extracted as previously described (Busov et al., 2003). Reverse transcription was performed on 1 μg of DNAase I-treated total RNA in a final reaction volume of 20 μl using an MMuLV (Moloney Murine Leukemia Virus) reverse transcriptase (ThermoFisher Scientific) following the manufacturer's protocol. Quantitative RT-PCR (qRT-PCR) was performed using the StepOnePlus Real Time System (Applied Biosystems) with the Maxima SYBR Green detection system (Thermo Fisher). Each PCR reaction contained 1× Maxima SYBR Green qPCR master mix, 0.1 μM of each forward and reverse primer (Eurofins MWG Operon), 1 μl of 10 × diluted cDNA solution and nuclease-free water. The final volume of each PCR reaction was 20 μl. The qRT-PCR cycling stages consisted of initial denaturation at 95°C for 10 min, followed by 40 cycles of 95°C for 15 s and 60°C for 1 min, and a final melting curve stage of 95°C for 15 s, 60°C for 1 min and 95°C for 15 s. qRT-PCR was performed with three biological and two technical replicates for each sample. Relative gene expression was calculated as previously described (Livak and Schmittgen, 2001; Schmittgen and Livak, 2008; Tsai et al., 2006; Yordanov et al., 2017). The *Ubq* gene expression was used as a loading control (Yordanov et al., 2010). All primers used in the gene expression analyses are shown in **Supplementary Table S2**. Primers were designed using the Primer-BLAST web resource at NCBI (National Center for Biotechnology Information; http://www.ncbi.nlm.nih.gov/BLAST).

### Cell wall analyses

Wood samples were milled to a 20-mesh using a Wiley mill. Approximately 4 mg of milled wood sample was measured and loaded into metal cups, which were then placed into an auto-sampler tray. The cell wall composition was analyzed by studying pyrolysis vapors produced using a commercially available molecular beam mass spectrometer (PyMBMS) designed specifically for biomass analysis as previously described (Sykes et al., 2009).

## Data Availability

The original contributions presented in the study are included in the article/**Supplementary Material**. Further inquiries can be directed to the corresponding author.
