# Supplementary material for: Discovery of genes that positively affect biomass and stress associated traits in poplar
Source: Front Plant Sci. 2024 Oct 18;15:1468905. doi: 10.3389/fpls.2024.1468905 (PMC11528158; doi:10.3389/fpls.2024.1468905)
Supplement: Supplementary file 1 [file Table1.docx]

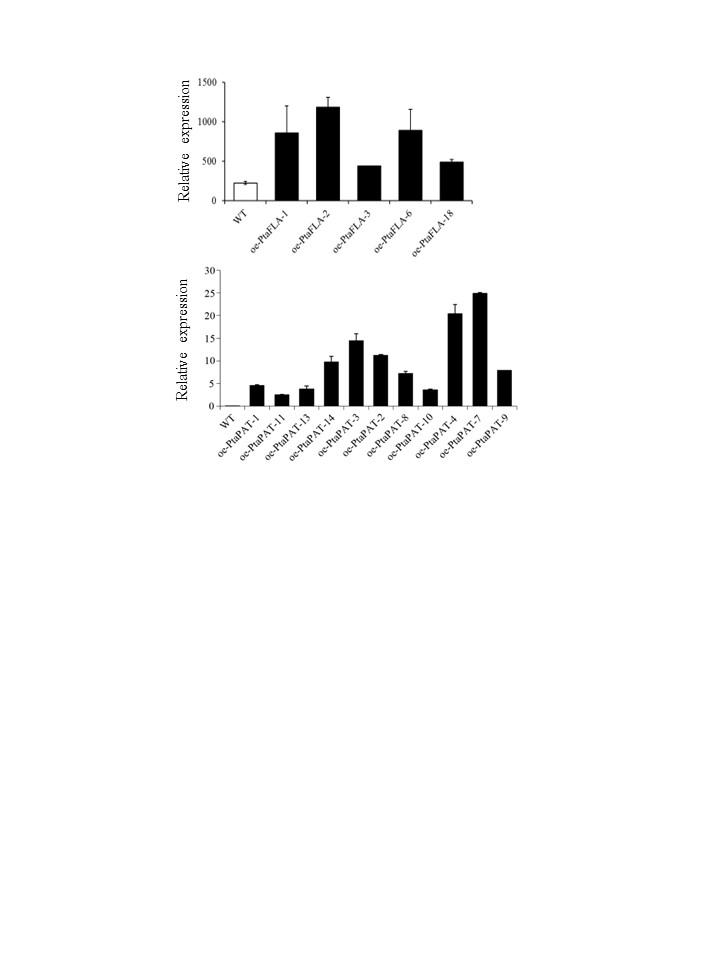


**Supporting Figure: Figure S1**. Validation of the transgenic up regulation of the candidate genes in independent recapitulation transgenic lines.

RNA extracted from plant leaves grown in control media and measured and analyzed gene expression using qRT-PCR. Values indicate mean ± SEM (*n* = 3). All lines are statistically different from WT-717 at least at *p* < 0.05 calculated using Student’s *t-*test.
