# Supplementary material for: Discovery of genes that positively affect biomass and stress associated traits in poplar
Source: Front Plant Sci. 2024 Oct 18;15:1468905. doi: 10.3389/fpls.2024.1468905 (PMC11528158; doi:10.3389/fpls.2024.1468905)
Supplement: Supplementary file 2 [file DataSheet1.pdf]

Table S1. Characterization of the T-DNA insertions in activation tagging lines with improved biomass traits. All insertions were mapped in the Populus genome using BLAST analysis tool in Phytozome v11.0 data base (https://phytozome.jgi.doe.gov).

Identifier #	Position (BLAST hit)	Identity (bp)	E-value	Left flanking loci / Distance from left border to start codon in Kbp [insertion = 6.7Kbp]		Right flanking loci / Distance from left border to start codon in Kbp [insertion = 6.7Kbp]		Activated/Most likely activated loci	Putative Gene function	Arabidopsis Homolog	Feature	Distance to gene (Kbp)
A630-7	Chr14:4,719,725..4,720,217	459/494	0.0E+00	PtXaTreH.14G052500	12.6	PtXaTreH.14G052600	0.6	PtXaTreH.14G052600	GROWTH-REGULATING FACTOR 9	AT2G45480.1	Intron	0.6
A541-1	Chr05:19,283,082..19,283,351	264/270	4.0E-127	PtXaAlbH.05G161200	24.3	PtXaAlbH.05G161300	5.4	PtXaAlbH.05G161300	TIC-LIKE PROTEIN	AT3G22380.1	Upstream region	5.4
A689-4	Chr10:17,990,503..17,991,103	589/601	0.0E+00	PtXaAlbH.10G146700	0.9	PtXaAlbH.10G146800	13.6	PtXaAlbH.10G146700	RIBONUCLEASE P SUBUNIT P38	AT1G13790.2	5' untranslated region (UTR)	0.9
A726-3	Chr14:9,913,312..9,913,644	327/333	2.0E-162	PtXaTreH.14G114700	17.3	PtXaTreH.14G114800	9.3	PtXaTreH.14G114800	ACTIN-LIKE ATPASE SUPERFAMILY PROTEIN	AT1G30540.1	Intron	9.3
A842-3	Chr10:10,630,097..10,630,565	460/470	0.0E+00	PtXaAlbH.10G086100	1.7	PtXaAlbH.10G086200	9.2	PtXaAlbH.10G086100	NO FUNCTIONAL ANNOTATION	AT3G07100.1	Upstream region	1.7
A82-2	Chr06:18,933,087..18,933,370	284/284	4.0E-144	PtXaTreH.06G160100	16.4	PtXaTreH.06G160200	3.8	PtXaTreH.06G160200	LARGE SUBUNIT RIBOSOMAL PROTEIN L21E (RP-L21E, RPL21)	AT1G57860.1	Upstream region	3.8
A835-3	Chr15:11,424,623..11,425,039	398/419	0.0E+00	PtXaTreH.15G090100	5.9	PtXaTreH.15G090200	6.9	PtXaTreH.15G090100	UNCHARACTERIZED	AT5G51150.1	Downstream region	5.9
A771-3	Chr08:711,508..711,709	201/202	4.0E-98	PtXaAlbH.08G010800	0.8	PtXaAlbH.08G010900	10.9	PtXaAlbH.08G010800	GLUTAREDOXIN FAMILY PROTEIN	AT5G01420.1	Upstream region	0.8
A826-3	Chr01:1,396,275..1,396,440	128/168	1.00E-22	PtXaTreH.01G014500	9.4	PtXaTreH.01G014600	20.8	PtXaTreH.01G014500	HELIX-LOOP-HELIX DNA-BINDING DOMAIN (HLH)	AT1G51140.1	Upstream region	9.4
A927-3	Chr06:7,429,340..7,429,974	477/581	2.0E-159	PtXaTreH.06G072700	0.6	PtXaTreH.06G072800	12.7	PtXaTreH.06G072700	ACYL-COA N-ACYLTRANSFERASE WITH RING/FYVE/PHD-TYPE ZINC FINGER DOMAIN-CONTAINING PROTEIN	AT2G37520.1	5' UTR	0.6
A972-5	Chr06:909,369..909,805	290/349	1.0E-99	PtXaTreH.06G010900	8.5	PtXaTreH.06G011000	11.2	PtXaTreH.06G010900	F-BOX PROTEIN CPR30	AT4G12560.1	Upstream region	8.5
A634-2	Chr08:4,458,012..4,458,561	550/550	0.0E+00	PtXaTreH.08G059200	0.0	PtXaTreH.08G059300	11.4	PtXaTreH.08G059200	(Z)-GAMMA-BISABOLENE SYNTHASE 1-RELATED	AT5G05350.1	Exon	0.0
A635-1	Chr13:1,946,091..1,946,362	260/276	1.0E-113	PtXaTreH.13G026900	17.4	PtXaTreH.13G027000	0.2	PtXaTreH.13G027000	TRANSPORT PROTEIN PARTICLE (TRAPP) COMPLEX SUBUNIT	AT3G05000.1	5' UTR	0.2
A822-3	Chr06:25,176,034..25,176,909	839/880	0.0E+00	PtXaAlbH.06G217500	9.8	PtXaAlbH.06G217600	8.1	PtXaAlbH.06G217600	SHN SHINE , DNA BINDING / TRANSCRIPTION FACTOR // ETHYLENE-RESPONSIVE TRANSCRIPTION FACTOR ERF003	AT5G25190.1	Upstream region	8.1
A862-1	Chr06:11,011,939..11,012,043	105/105	4.0E-47	PtXaAlbH.06G097000	11.9	PtXaAlbH.06G098000	0.6	PtXaAlbH.06G098000	NO FUNCTIONAL ANNOTATION	N/A	Upstream region	0.6
A863-3	Chr08:1,237,541..1,237,795	253/255	1.0E-125	PtXaAlbH.08G019100	0.2	PtXaAlbH.08G019200	13.2	PtXaAlbH.08G019100	OTU DOMAIN CONTAINING PROTEIN	AT3G57810.4	5' UTR	0.2
A836-1	Chr18:9,569,679..9,570,034	350/359	1.0E-170	PtXaAlbH.18G066300	19.0	PtXaAlbH.18G066400	2.0	PtXaAlbH.18G066400	HOMOGENTISATE PHYTYLTRANSFERASE / HPT	AT2G18950.1	Intron	2.0
A857-2	Chr01:36,423,209..36,423,290	74/82	8.0E-23	PtXaAlbH.01G282600	1.3	PtXaAlbH.01G282700	27.8	PtXaAlbH.01G282600	E3 UBIQUITIN-PROTEIN LIGASE RGLG1-RELATED	AT5G14420.1	Exon	1.3
A885-1	Chr10:17,452,998..17,453,190	193/193	7.0E-95	PtXaAlbH.10G139600	6.3	PtXaAlbH.10G139700	7.7	PtXaAlbH.10G139600	ZINC FINGER CCCH DOMAIN-CONTAINING PROTEIN 37-RELATED	AT3G12680.1	3' UTR	6.3
A934-2	Chr18:142,446..142,543	96/98	7.0E-41	PtXaAlbH.18G001300	0.2	PtXaAlbH.18G001400	18.0	PtXaAlbH.18G001300	SERINE PROTEASE FAMILY S1C HTRA-RELATED	AT5G27660.2	Exon	0.2
A943-1	Chr04:2,205,837..2,205,924	82/88	7.0E-30	PtXaAlbH.04G022700	6.5	PtXaAlbH.04G022800	11.7	PtXaAlbH.04G022700	KINESIN-1-RELATED	AT4G05180.1	Upstream region	6.5
A991-1	Chr01:38,738,885..38,739,199	312/315	3.0E-156	PtXaTreH.01G300500	0.1	PtXaTreH.01G300600	11.8	PtXaTreH.01G300500	MEIOTIC RECOMBINATION PROTEIN SPO11	AT3G13170.1	5' UTR	0.1
B21-1	Chr11:15,661,025..15,661,305	247/281	1.7E-88	PtXaAlbH.11G081800	5.8	PtXaAlbH.11G081900	8.7	PtXaAlbH.11G081800	UBIQUITIN CARBOXYL-TERMINAL HYDROLASE 36/42	AT3G14400.1	Downstream region	5.8
A613-2	Chr01:48,013,773..48,014,052	229/281	2.0E-68	PtXaAlbH.01G351100	12.2	PtXaAlbH.01G351200	12.8	PtXaAlbH.01G351100	BOI-RELATED E3 UBIQUITIN-PROTEIN LIGASE 3-RELATED	AT3G12920.1	Upstream region	12.2
A874-9	Chr02:17,294,793..17,294,986	193/194	2.0E-93	PtXaTreH.02G173800	8.5	PtXaTreH.02G173900	16.3	PtXaTreH.02G173800	DIMETHYLANILINE MONOOXYGENASE	AT1G48910.1	Upstream region	9.6
A901-5	Chr12:8,571,009..8,571,304	239/298	1.0E-66	PtXaTreH.12G059500	25.1	PtXaTreH.12G059600	1.8	PtXaTreH.12G059600	GDSL-MOTIF LIPASE/HYDROLASE FAMILY PROTEIN	AT5G62930.1	Intron	1.8
B0-4	Chr04:811,140..811,512	373/373	0.0E+00	PtXaTreH.04G008500	2.6	PtXaTreH.04G008600	12.7	PtXaTreH.04G008500	GLYOXALASE I HOMOLOG	AT3G53700.1	Upstream region	2.6
A915-2	Chr14:5,531,694..5,531,727	31/34	1.6E-03	PtXaAlbH.14G064200	0.2	PtXaAlbH.14G064300	22.1	PtXaAlbH.14G064200	PHOSPHATIDYLINOSITOL N-ACETYGLUCOSAMINLYTRANSFERASE SUBUNIT P-LIKE PROTEIN	AT4G00440.3	Intron	0.2
A975-4	Chr10:7,703,668..7,704,334	388/388	0.0E+00	PtXaAlbH.10G054200	0.3	PtXaAlbH.10G054300	28.9	PtXaAlbH.10G054200	TOPP4, TYPE ONE SERINE/THREONINE PROTEIN PHOSPHATASE 4	AT2G39840.1	Upstream region	0.3
A955-1	Chr07:881,027..881,480	445/454	0.0E+00	PtXaTreH.07G010100	14.1	PtXaTreH.07G010200	1.4	PtXaTreH.07G010200	U-BOX DOMAIN-CONTAINING PROTEIN 50-RELATED	AT5G65500.1	Exon	1.4
A979-4	Chr01:17,597,610..17,597,872	263/263	8.9E-133	PtXaAlbH.01G162300	65.1	PtXaAlbH.01G162400	1.2	PtXaAlbH.01G162400	ABC TRANSPORTER G FAMILY MEMBER 35-RELATED	AT1G59870.1	Exon	1.2
199p-5	Chr12:10,989,445..10,989,698	226/254	1.4E-85	PtXaAlbH.12G082300	7.1	PtXaAlbH.12G082400	9.2	PtXaAlbH.12G082300	PROTEIN MEI2-LIKE 1-RELATED	AT5G61960.3	3' UTR	7.1
795L-6	Chr06:3,283,1..3,340,4	140/149	1.9E-58	PtXaTreH.06G000100	0.0	PtXaTreH.06G000200	13.2	PtXaTreH.06G000100	PENTATRICOPEPTIDE REPEAT-CONTAINING PROTEIN	AT5G03560.4	Exon	0.0
826L-3	Chr08:3,531,725..3,531,969	245/245	5.3E-123	PtXaAlbH.08G048500	13.0	PtXaAlbH.08G048600	3.0	PtXaAlbH.08G048600	WALL-ASSOCIATED RECEPTOR KINASE GALACTURONAN-BINDING DOMAIN	AT5G02070.1	Upstream region	3.0
345L-1	Chr10:16,317,536..16,317,975	440/440	0.0E+00	PtXaAlbH.10G123500	17.4	PtXaAlbH.10G123600	3.8	PtXaAlbH.10G123600	NUCLEAR TRANSPORT FACTOR 2 (NTF2) FAMILY PROTEIN	AT1G13730.2	3' UTR	3.8
501L-5	Chr14:1,957,182..1,957,297	114/125	8.6E-44	PtXaTreH.14G021900	17.7	PtXaTreH.14G022000	0.0	PtXaTreH.14G022000	MEMBRANE PROTEIN YCF1-RELATED	ATMG00370.1	Exon	0.0
575L-1	Chr01:34,466,030..34,466,136	107/107	2.5E-48	PtXaAlbH.01G268000	2.0	PtXaAlbH.01G268100	16.6	PtXaAlbH.01G268000	DOUBLE-STRANDED RNA-BINDING PROTEIN 3	AT3G26932.1	3' UTR	2.0
659L-1	Chr06:23,150,863..23,151,505	642/643	0.0E+00	PtXaTreH.06G205900	3.5	PtXaTreH.06G206000	9.1	PtXaTreH.06G205900	HISTONE H2A (H2A)	AT1G52740.1	Upstream region	3.5
707L-1	Chr01:33,916,483..33,916,815	331/333	5.5E-168	PtXaTreH.01G268700	24.4	PtXaTreH.01G268800	7.2	PtXaTreH.01G268800	HYPOTHETICAL PROTEIN	AT3G01960.1	Upstream region	7.2
994L-1	Chr14:4,373,677..4,374,201	482/553	1.8E-172	PtXaTreH.14G048000	26.7	PtXaTreH.14G048100	3.8	PtXaTreH.14G048100	AUXIN-REGULATED PROTEIN-RELATED	AT3G60690.1	Upstream region	3.8
