# Supplementary material for: Discovery of genes that positively affect biomass and stress associated traits in poplar
Source: Front Plant Sci. 2024 Oct 18;15:1468905. doi: 10.3389/fpls.2024.1468905 (PMC11528158; doi:10.3389/fpls.2024.1468905)
Supplement: Supplementary file 3 [file DataSheet2.pdf]

Table S2. Primers used for the real-time RT-PCR analysis of the genes flanking the T-DNA insertion site.

Gene Name	Primer 5'-3' sequence
PtXaTreH.14G052500	**Fwd**-TTGGCTAGTAACGGTGCAGG<br>**Rev**-TCAACCGGTAGCAGCACATT
PtXaTreH.14G052600	**Fwd**-TGGATCCAGAACCTGGGAGA<br>**Rev**-TGAGGTGATGGTTTTTCAGAAGC
PtXaAlbH.05G161200	**Fwd**-CGCACTGTGAAACCACCAAG<br>**Rev**-CAAGGTGGATGAGAGCAGCA
PtXaTreH.01G014500	**Fwd**-ACAGGGGACTAAATGGAGCAC<br>**Rev**-ACTCGGTGCGGATTGATACC
PtXaAlbH.10G146700	**Fwd**-AAGAGCAGCCTTTTGTCCCC<br>**Rev**-TGAGCCACTTTCCCCAACAC
PtXaAlbH.08G010800	**Fwd**-GAGGGGCTGAAAAGATGAAAGG<br>**Rev**-CCTGCGCTTCGAGACTAGG
PtXaAlbH.10G086200	**Fwd**-GGAACAACTGCCTTGCTGAC<br>**Rev**-TCCAATTTCCAATCCCCGACA
PtXaTreH.15G090200	**Fwd**-GCAGCAGCAATGCTCACTTT<br>**Rev**-ACGCGGTTCCATAAGTACCA
PtXaTreH.06G072700	**Fwd**-AGCGTAATCCATTTCCGGTGA<br>**Rev**-ACGCATTCGTAGGCGATTCT

Table S3. Primers used for cloning of the candidate genes following Gateway procedure.

**Gene Name**	***att*B primer 5'-3' sequence**
PtXaTreH.14G052500	**Fwd-** GGGGACAAGTTTGTACAAAAAAGCAGGCT atggctacgttacagtactctctcc
	**Rev-** GGGGACCACTTTGTACAAGAAAGCTGGGT tcaggacatgagaatcgaaca
PtXaTreH.14G052600	**Fwd-** GGGGACAAGTTTGTACAAAAAAGCAGGCTatggagaaaagagtatctga
	**Rev-** GGGGACCACTTTGTACAAGAAAGCTGGGTctaagaagaaaggtttttgtt
PtXaAlbH.05G161200	**Fwd-** GGGGACAAGTTTGTACAAAAAAGCAGGCTatggaccttagtaaggcaacgctagagatc
	**Rev-** GGGGACCACTTTGTACAAGAAAGCTGGGTttagcgactgttggcaagaggactaacggc
